# Fungal Keratitis: Epidemiology, Rapid Detection, and Antifungal Susceptibilities of* Fusarium* and* Aspergillus* Isolates from Corneal Scrapings

**DOI:** 10.1155/2019/6395840

**Published:** 2019-01-20

**Authors:** Palanisamy Manikandan, Ahmed Abdel-hadi, Yendrembam Randhir Babu Singh, Rajaraman Revathi, Raghavan Anita, Saeed Banawas, Abdul Aziz Bin Dukhyil, Bader Alshehri, Coimbatore Subramanian Shobana, Kanesan Panneer Selvam, Venkatapathy Narendran

**Affiliations:** ^1^Department of Medical Laboratory Sciences, College of Applied Medical Sciences, Majmaah University, Al Majmaah, Saudi Arabia; ^2^Aravind Eye Hospital and Postgraduate Institute of Ophthalmology, Coimbatore, India; ^3^Greenlink Analytical and Research Laboratory India Private Limited, Coimbatore, India; ^4^Botany and Microbiology Department, Faculty of Science, Al-Azhar University, Assiut Branch, Egypt; ^5^Microbial Resources Division, Institute of Bioresources and Sustainable Development, Takyel, Imphal, Manipur, India; ^6^Department of Microbiology, PSG College of Arts and Science, Coimbatore, India; ^7^Department of Microbiology, M.R Government Arts College, Mannargudi, Tamilnadu, India

## Abstract

Fungal aetiology of keratitis/corneal ulcer is considered to be one of the leading causes of ocular morbidity, particularly in developing countries including India. More importantly,* Fusarium* and* Aspergillus *are reported commonly implicating corneal ulcer and against this background the present work was undertaken so as to understand the current epidemiological trend of the two fungal keratitis. During the project period, a total of 500 corneal scrapings were collected from suspected mycotic keratitis patients, of which 411 (82.2%) were culture positive for bacteria, fungi, and parasites. Among fungal aetiologies,* Fusarium *(216, 52.5% of 411) and* Aspergillus *(68, 16.5% of 411) were predominantly determined. While the study revealed a male preponderance with both the fungal keratitis , it further brought out that polyene compounds (natamycin and amphotericin B) and azoles were active, respectively, against* Fusarium* spp. and* Aspergillus *spp. Additionally, 94.1% of culture proven* Fusarium *keratitis and, respectively, 100% and 63.6% of* A. flavus* and* A. fumigatus* were confirmed by multiplex PCR. The sensitivity of the PCR employed in the present study was noted to be 10 fg/*μ*l, 1 pg/*μ*l, and 300 pg/*μ*l of DNA, respectively, for* Fusarium*,* A. flavus,* and* A. fumigatus.* Alarming fact was that* Fusarium *and* Aspergillus *regionally remained to be the common cause of mycotic keratitis and the* Fusarium *isolates had a higher antifungal resistance than* Aspergillus* strains against most of the test drugs.

## 1. Introduction

Blindness due to corneal infections is a serious problem next to cataract [1] and fungal infections of the cornea have emerged as a major eye disease globally. The corneal infection of fungal etiology is very common and comprising at least 50% of all culture positive cases in India [2]. However, the prevalence rate varies from one country to the other and also from one population to another within the same country [3, 4]. In South India, the dominance of fungal keratitis, particularly of* Fusarium* and* Aspergillus, *is prevalent more than a decade and have been documented in many literatures [5–8]. Species of* Fusarium* and* Aspergillus* are widespread in nature being causative agents of important diseases of major crops as well as immunocompromised humans [9] and have been considered as important pathogens in eye infections, especially keratitis [2, 10]. In India,* Fusarium* and* Aspergillus* species are being isolated from corneal ulcers in large numbers [6, 8, 11–14], irrespective of the geographical location.

The importance of fungal keratitis gained momentum in 2005 following the outbreak of fungal keratitis among contact lens wearers in many developed countries [15, 16]. The fact that the outcome of fungal keratitis is worse than that of bacterial keratitis must be underscored [17] due to poor response to the therapy as well as the limited availability of antifungal agents [18]. The diagnosis and treatment of fungal keratitis is one of the most difficult problems encountered by ophthalmologists. Further, the prognoses of the fungal keratitis worsen with inadvertent antifungal agents and recalcitrant course of the disease [2]. Although voriconazole and other triazoles have broad-spectrum activity against causative fungal isolates, clinically no single drug was found to be effective against fungal keratitis. Also,* Fusarium* spp. are completely tolerant to itraconazole and caspofungin [19].

Accurate identification of the aetiological agent of fungal keratitis is of great importance in order to administer appropriate treatment [10, 20]. Though conventional culture methods are often useful, it takes more time for sufficient growth and subsequent identification of the causative agent [2]. The use of molecular techniques offers a significant reduction in time required for precise diagnosis of such infections [20, 21]. Also, the scarcity of region-specific antifungal susceptibility data, the limited availability of commercially available antifungal drugs, and the lack of response lead to corneal blindness in a high number of infected patients. Therefore, due to the magnitude of the fungal keratitis in Tamilnadu, India, a survey of local antifungal susceptibility pattern, and exploring a suitable, rapid diagnostic method is of paramount importance.

Hence, the present study was designed for the rapid detection fungal pathogens causing keratitis by multiplex polymerase chain reaction (PCR) and also to evaluate the efficacy of multiplex PCR against routine culture method. Further, the study also presents data on regional prevalence of fungal keratitis and minimum inhibitory concentration [4] values of routinely used antifungal agents against* Fusarium* and* Aspergillus *isolated from corneal ulcers. The paper not only provides information on the current incidence of* Fusarium/Aspergillus *keratitis but also gives valuable information on drug susceptibilities so as to help the ophthalmologists to initiate appropriate antifungal regimen against fungal keratitis.

## 2. Materials and Methods

### 2.1. Patients

A total of 500 (including repeat specimens) corneal scraping specimens were collected between June 2010 and January 2011 from clinically suspected patients with mycotic keratitis who attended Cornea services at Aravind Eye Hospital, Coimbatore, after obtaining ethical clearance (Institutional Review Board, Aravind Medical Research Foundation, Madurai, India).

### 2.2. Collection of Specimens

Corneal scraping was performed under aseptic condition by an ophthalmologist using a sterile Kimura's spatula and a portion was inoculated on 5% sheep blood agar (SBA), chocolate agar (CA), and potato dextrose agar (PDA). Additionally, the remaining specimen was smeared on two clear glass slides to observe the presence of fungal filaments microscopically using 10% KOH wet mount and Gram staining. Corneal scrapings that revealed fungal filaments in direct microscopy were considered for the study. Repeat corneal scraping was done to collect specimen for PCR assay. The collected specimens were placed in 400 *μ*l of lysis buffer (0.5M Tris HCL, 0.5M EDTA, 3% SDS, 1%  *β*-mercaptoethanol) and were stored at −20°C until further processing.

### 2.3. Identification of* Fusarium* spp. and* Aspergillus* spp. by Culture Method

All fungal isolates were identified based on the standard culture techniques followed by microscopy after lacto-phenol cotton blue staining. The identified isolates were preserved in 0.85% saline at 4°C.

### 2.4. Determination of Minimum Inhibitory Concentration (MIC)

The MICs of seven different antifungal agents, namely, amphotericin B (Himedia, Mumbai, India), itraconazole (Sigma-Aldrich, St. Louis, MO, USA), natamycin (Sigma-Aldrich, St. Louis, MO, USA), voriconazole (Aurolab, Madurai, India), ketoconazole (Himedia, Mumbai, India), econazole (Aurolab, Madurai, India) and clotrimazole (Aurolab, Madurai, India) were determined in accordance with the guidelines of Clinical and Laboratory Standards Institute (CLSI) [22]. The MIC values were defined as the lowest concentrations of antimicrobials that inhibit the visible growth of an isolate. The MIC_50_ and MIC_90_ values were defined as the MICs required to inhibit the growth of 50% and 90% of the isolates from a given species, respectively [23].

*A. flavus* ATCC 204304 was included as a quality control strain in all the batches of MIC analysis. The antifungal agents were prepared in order to achieve the dilution ranges in the order of 8 *μ*g/ml - 0.015* μ*g/ml (amphotericin B, econazole, voriconazole, and clotrimazole), 32* μ*g/ml - 0.06 *μ*g/ml (itraconazole), 16 *μ*g/ml - 0.03* μ*g/ml (ketoconazole), and 128 *μ*g/ml - 0.25* μ*g/ml (natamycin).

### 2.5. DNA Extraction

DNA was extracted from infected corneal tissue scrapings using Qiagen DNA extraction kit (Hilden, Germany), as per the manufacturer instructions. The concentration of extracted DNA was determined by nanophotometer (Implen, Munich, Germany). The DNA was stored at −20°C (Sanyo, Osaka, Japan) until further use.

### 2.6. Polymerase Chain Reaction

The extracted DNA was initially subjected to the first round of PCR using universal fungal primers (*ITS1* and* ITS4*, internal transcribed spacer region). The first round amplicons were subsequently subjected for multiplex PCR using* Fusarium *and* Aspergillus *specific primers [24].

For each reaction in the first round PCR, a cocktail comprising 10 *μ*l of DNA extract with 5 *μ*l of 10 × PCR buffer (mixed with 1.5 mM magnesium chloride), 1 *μ*l of dNTPs mix (200 *μ*M each dNTPs), 20 pm/*μ*l of each primer, and 0.5 U of Taq DNA polymerase amounting to a total volume of 50 *μ*l was prepared. The* ITS* primers (Sigma, St Louis, MO, USA) used were 5′-TCCGTAGGTGAACCTGCGG-3′ (F) and 5′-TCCTCCGCTTATTGATATGC-3′ (R). The reaction was run in gradient thermocycler (Eppendorf, Hamburg Germany) involving initial denaturation at 95°C for 5 min, followed by 34 cycles in series of denaturation at 95°C for 30s, annealing at 54°C for 1 min, and extension at 72°C for 1 min, with a final step of extension at 72°C for 6 min and final holding at 4°C.

Multiplex PCR cocktail was prepared as described above. The specific primers (Sigma, St Louis, MO, USA) used for identification of* Fusarium* spp.,* A. flavus, *and* A. fumigatus* were CAACTCCCAAACCCCTGTGA (F) & GCGACGATTACCAGTAACGA (R), CCGCCGGAGACACCACGAAC (F) & TGGGCAGCAATGACGCTCGG (R), and TTGTGTGTTGGGCCCCCGTC (F) & AAAGTTGGGTGTCGGCTGGCG (R), respectively. The amplicons were subjected to agarose gel electrophoresis in 1.5% agarose (Sigma, St. Louis, MO, USA) for 20-25 min at 80V (GeneI, Bangalore, India) along with 100 bp (Sigma, St. Louis, MO, USA) molecular marker. The DNA bands were visualized, analyzed, and documented using gel documentation system (Vilber Lourmat, France).

## 3. Results

Of the 500 corneal scrapings collected during the study period, the culture revealed that 411 (82.2% of 500) were positive for fungi, bacterial, and mixed etiologies (Table 1). Of 402 ocular specimens (97.8% of 411), 6 (1.4% of 411) and 3 (0.72% of 411) were identified to be due to fungal, bacterial, and mixture of bacterial and fungal causes, respectively. Further, 10% KOH and Gram staining revealed that 96.1% (449 of 467) and 94.7% (473 of 499) correlated with culture findings in the detection of fungi from corneal scrapings. Number of* Fusarium *(n=216) keratitis cases occurred more in males (134, 62%) than among females (82, 38%). The age group affected with* Fusarium* keratitis ranged from 21 to 70 years and particularly, 66 patients (30.5% of 216) belonged to 41-50 years and 50 (23.1% of 216) belonged to 51-60 years.

Similarly,* Aspergillus *keratitis was confirmed predominantly among males (55.8% of 68). The age group affected with* Aspergillus* keratitis ranged from 31 to 50 years (31, 45.5% of 68).* A. flavus* (48, 11.6% of 411) was the predominant species among the identified* Aspergillus *identified during the study period.* Bipolaris *spp. (22, 5.3% of 411),* Curvularia* spp. (12, 2.9% of 411), and* Exserohilum* spp. (11, 2.6% of 411) were the other fungi isolated during the study period.

### 3.1. Minimum Inhibitory Concentration (MIC)

In this study, a total of 200* Fusarium* and 67* Aspergillus* isolates (47* A. flavus*, 11* A. fumigatus*, 5* A. terreus*, 3* A. niger,* and 1* A. tamarii*) were included to determine the minimum inhibitory concentrations / MIC_50_ and MIC_90_ of routine antifungal drugs. Overall, the isolates of* Fusarium* spp. required higher concentrations (Tables 2 and 3) of specific antifungal drug than* Aspergillus* spp. in order to be inhibited. Most of the* Aspergillus* isolates were inhibited by amphotericin-B at a concentration of ≥ 1 *μ*g/ml. More interestingly, natamycin acted against 65% (130, n = 200) of the* Fusarium* isolates at a concentration of 16 *μ*g/ml while majority of* Aspergillus* spp. were inhibited at ≥ 32 *μ*g/ml. A notable observation was with itraconazole activity, where 92.5% (185, n = 200)* Fusarium* spp. were susceptible only at a concentration of ≥ 32 *μ*g/ml while 100% (n = 67) of the* Aspergillus* isolates were completely inhibited at ≤ 1 *μ*g/ml. Similar MIC patterns were observed with econazole, clotrimazole, and ketoconazole where* Fusarium* isolates had higher MICs compared to* Aspergillus* spp.

### 3.2. PCR Study

A total of 473 corneal scrapings which were culture positive for* Fusarium* spp. (205),* A. flavus* (46),* A. fumigatus* (11),* A. terreus* (4),* A. tamarii* (1),* Bipolaris* spp. (22),* Exserohilum* spp. (10),* Curvularia *spp. (12),* Cladosporium *spp. (4),* Aureobasidium* spp. (3),* Exophiala* spp. (2),* Lasiodiplodia* sp. (1),* Pseudallescheria* sp. (1),* Alternaria* sp. (1),* Scedosporium *spp. (2), UID (20), and UIH (36) were included for PCR. Additionally, 86 culture negative corneal scrapings along with 2 mixed infections and 4 bacterial positive specimens were also included for PCR analysis.

**Figure 1:**
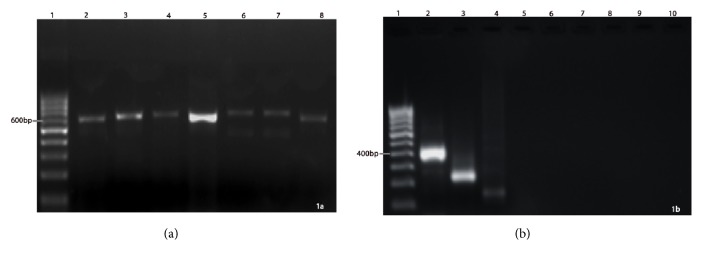
First and second round of PCR amplifications (a) Uniplex PCR with ITS1 & ITS4 primers: Lane 1 - 100 bp ladder, lane 2 -* Fusarium* sp. (∼600 bp), lane 3 -* A. flavus*, lane 4 -* A. fumigatus*, lane 5 -* Bipolaris* spp., lane 6 -* Exerohilum* sp., lane 7 -* Alternaria* sp., lane 8 -* Curvularia* sp. (b) Multiplex PCR with species specific primers: Lane 1 - 100 bp marker, Lane 2 -* Fusarium* sp., Lane 3 -* A. flavus*, Lane 4 -* A. fumigatus*, Lane 5 -* Bipolaris* sp., Lane 6 -* Exerohilum* sp., Lane 7 -* Alternaria* sp., Lane 8 -* Curvularia* sp. Lane 9 - UID, Lane 10 - UID.

All the primers specifically amplified the target region. The specific primers of ITS 1 & 4 (1st round),* Fusarium* spp.,* A. flavus*, and* A. fumigatus* after PCR and upon electrophoresis produced amplicons of approximately 600 bp, 400 bp, 250 bp, and 150 bp, respectively (Figure 1(a)). In addition, other fungal culture positive corneal scrapings such as* Bipolaris, Curvularia, Exserohilum,* etc. could not be amplified in 2nd round multiplex PCR (Figure 1(b)). All the PCR positive specimens (*Fusarium, A. flavus, A. fumigatus*) were further confirmed with culture identification to ensure the specificity.

### 3.3. Sensitivity of the PCR

**Figure 2:**
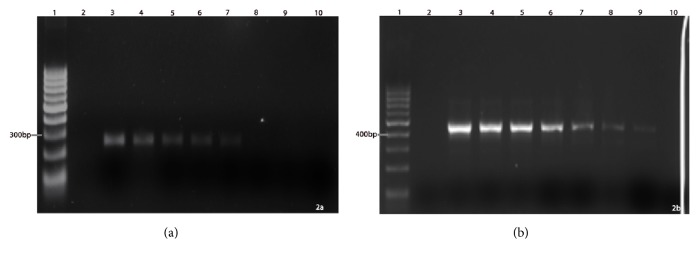
Determination of PCR sensitivity and specificity (a)* A. flavus*: Lane 1 - 100 bp marker, lane 2 - negative control, Lane 3 - 10 ng, Lane 4 - 1 ng, Lane 5 - 100 pg, lane 6 - 10 pg, lane 7 - 1 pg, lane 8 - 100 fg, lane 9 - 10 fg, lane 10 - 1 fg. (b)* Fusarium* sp.: Lane 1 - 100 bp marker, lane 2 - negative control, Lane 3 - 10 ng, Lane 4 - 1 ng, Lane 5 - 100 pg, lane 6 - 10 pg, lane 7 - 1 pg, lane 8 - 100 fg, lane 9 - 10 fg, lane 10 - 1 fg.

**Figure 3:**
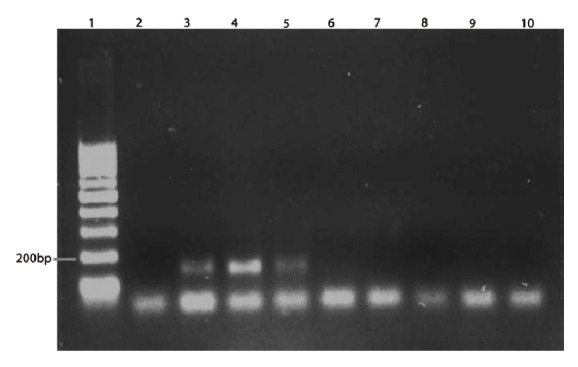
PCR sensitivity for* A. fumigatus*. Lane 1 - 100 bp marker, lane 2 - negative control, lane 3 - 30 ng, lane 4 - 3 ng, lane 5 - 300 pg, lane 6 - 30 pg, lane 7 - 3 pg, lane 8 - 300 fg, lane 9 - 30 fg, lane 10 - 3 fg.

To determine the minimum amount of fungal DNA that could be detected by the established PCR assay, variable quantities (ranging from 10 ng/*μ*l to 300 fg/*μ*l) of* Fusarium *and* Aspergillus *genomic DNA were used as DNA template (Figure 2(a)) and it was found that the best optimized PCR conditions could amplify* Fusarium *DNA as less as 10 fg /*μ*l (Figure 2(b)). Similarly,* A. flavus *DNA could be amplified as low as 1 pg/*μ*l. However, the PCR was noted to be less sensitive towards the detection of* A. fumigatus *DNA as it required a minimum DNA concentration of at least 300 pg/*μ*l for amplification and detection (Figure 3).

### 3.4. PCR-First Round of Amplification with ITS Primers

In the 1^st^ round of PCR amplification, using the universal fungal primers (ITS1 and ITS4), PCR products (550-600 bp) were generated from 347 (73.3%) corneal specimens. Of 347, 337 (97.1%) specimens were already confirmed to be fungal culture positive. An increase in the total number of positive cases through the applications of PCR under the project indicated the obvious and inevitable requirement of such techniques in routine diagnostic procedures. Also, the accuracy of the PCR detection was superior as most (16%, 76 of 473) of the PCR negative cases (ITS1 and ITS4) were also negative for conventional culture primarily. However, the DNA from 50 (10.5% of 473) corneal scrapings which were actually identified to be culture positive failed to amplify ITS 1 & 4 by PCR.

### 3.5. PCR-Second Round of Amplification with Fungal Species Specific Primers

The findings upon second round of amplification using exclusive primers for each genus/species were diverse. Of the 205 corneal scrapings that were positive for* Fusarium *spp. culture, only 193 (94.1% of 205) were reconfirmed as* Fusarium *spp. (Figure 1(b)). Similarly, 46 (100%)* A. flavus *and 7 (63.6% of 11)* A. fumigatus *were confirmed with PCR. No amplification of other fungal DNA was observed for which specific primers were not used (but their DNA could be amplified in the first round using universal fungal primers for ITS). More remarkably, of the 86 culture negative corneal scrapings, 9 (10.4% of 86) of them showed positive for* Fusarium *spp. in PCR which indicated that the PCR primers could identify even those specimens/cases which were reported to be negative in culture and that the primers could amplify minimum quantity of* Fusarium *DNA in culture negative cases also.

## 4. Discussion

Rapid identification of fungal pathogens and instilling of appropriate antifungal agents are key factors of a successful fungal keratitis management and the present study focused on the two features with special reference to* Fusarium *spp. and* Aspergillus* spp. The study included only fungal positive specimens identified through direct microscopy to evaluate the PCR specificity of rapid detection. The incidences of fungal keratitis reported were highly variable across the Indian states: southern and western India with 36.7% [8] and 36.3% [8, 25], northern (7.3%), northeastern (25.6%), and eastern India (26.4%) [26–28]. The present study revealed a direct microscopic sensitivity of 10% KOH and gram staining 96.1% and 94.7%, respectively, from corneal scrapings and were in accordance with Bibhudutta* et al*., 2011 [28]. Similarly, Bharathi* et al.* [8] reported 99.23% and 88.73% sensitivity in KOH wet mount and Gram staining, respectively. In another study, giemsa stain (75%) and Gram stain (55.5%) were used for the detection of fungal filaments [29].

Similar to other studies [8, 30], male patients (60.5%) were dominant with* Fusarium* and* Aspergillus *keratitis, than females (39.4%). Gonzales* et al.* [31] and Srinivasan* et al.* [6] reported the ratio of male to female with corneal ulcer as 1.6 to 1.* Fusarium* and* Aspergillus* keratitis were majorly (94%) confirmed in middle aged (21-70 years) individuals with a focused predominance in 41-50 years (28.8%).

Middle age group was noted to be highly vulnerable for fungal keratitis in Madurai [6] region with 31-60 and Nepal [32] with 21 -50 years. In this study, male patients were at a higher risk (55.8%) for* Aspergillus *keratitis, though our previous assessment [23] brought out 60% with 1.5:1 male to female ratio. In this study,* Fusarium *spp. (52.5%) followed by* Aspergillus* spp. (16.5%) were predominantly responsible for mycotic keratitis. Similar findings were observed in other parts of Southern India [6, 8] western India [25, 30], and eastern India [28]. Also, the prevalence of* Aspergillus *keratitis was found to be consistent with our previous findings [23]. However,* Aspergillus* spp. had been the dominant aetiology in fungal keratitis followed by* Fusarium* spp. in parts of northern [26] and eastern India [4]. In addition, the fungal keratitis aetiology greatly varied from country to country [10].* Candida *spp. with incidence rates of 60.6% and 32.7% were observed in London [33] and Melbourne [18], respectively.* Acremonium *spp. (40%) were the most predominant fungal isolate in Paraguay [34]. Antifungal susceptibility testing and MIC determination procedures have very significant role in terms of successful management of fungal keratitis patients. However, the limited availability of commercial antifungal agents especially in the form of eye drops made the therapy more complicated [10]. In this study,* Fusarium* spp. required higher concentration of antifungal agents to inhibit the growth when compared to* Aspergillus* spp. except for amphotericin B and natamycin. In a similar investigation, amphotericin-B and natamycin were reported with significant activity against* Fusarium* spp. [35]. Exactly, 90% of the* Fusarium* spp. were sensitive at 1 *μ*g/ml while 90% of* A. flavus, A. fumigatus, A. terreus,* and* A. niger *were sensitive at 2, 4, 2, and 0.5 *μ*g/ml, respectively [36]. However, an assessment by Lalitha* et al.* [19] and Isabel* et al.* [35] reported MIC_90_ at 4 *μ*g/ml and 4.62 *μ*g/ml, respectively, against* Fusarium* spp. with amphotericin-B. The MIC_90_ of* Aspergillus* spp. observed in the present study was similar to the study by Lalitha* et al.* [19]. Natamycin, though the drug of choice against filamentous fungi [37], because of its poor penetration, is effective only in nonsevere superficial keratitis [13].* Fusarium* spp. were more sensitive to natamycin than* Aspergillus *spp. In this study, 90% of the* Fusarium* and* Aspergillus *strains were inhibited at 32 *μ*g/ml and 64 *μ*g/ml, respectively, and the findings were similar to the previous assessments [19]. The resistant pattern of* Fusarium* spp. against itraconazole in this study was clearly evident from other reports [19, 35] though the MIC values showed variations. On the contrary,* Aspergillus *was significantly sensitive against itraconazole with consistent findings to our previous work [23] as well as with other investigators [19, 38]. Similar to itraconazole, other agents such as voriconazole, econazole, clotrimazole, and ketoconazole were relatively effective against* Aspergillus* spp. Likewise, higher drug concentrations were required to inhibit* Fusarium* spp. indicating that the tested azole drugs were ineffective. Eduardo* et al.* 2008 [39] and Lalitha* et al.* 2007 [19] reported MIC_90_ of voriconazole as 4 *μ*g/ml and the similar range was found in the present study (8 *μ*g/ml). Eduardo* et al.* concluded that* F. solani* tends to be more resistant to certain azoles [39]. However, in case of* Aspergillus *spp., highest MIC was noted against voriconazole (1 *μ*g/ml), which was similar to the data published previously [19, 23]. In case of ketoconazole, the MIC_90_ and MIC_50_ of* Fusarium* spp. were noted as 16 *μ*g/ml, while among* Aspergillus *spp.* A. flavus, A. terreus* and* A. niger *had a highest MIC_90_ of 4 *μ*g/ml. Isabel* et al.* 1997 [35] reported higher MIC_90_ (>51.20 mg/l) against* Fusarium. * On the contrary, Theresa* et al.* 2006 [36] reported higher MIC percentile value of ketoconazole in* A. niger* and* A. terreus* when compared to other filamentous fungi. In general, the isolates of* Fusarium* spp. showed more resistance than* Aspergillus* spp. Most of the* Fusarium* isolates required higher concentration of drugs to get inhibited. Further, most of the* Aspergillus* isolates were sensitive to amphotericin-B.

All the test PCR primers (ITS 1 & 4) amplified the target region of* Fusarium* spp.,* A. flavus*, and* A. fumigatus* representing 600 bp, 400 bp, 250 bp, and 150 bp, respectively, after fractionation and were confirmed with suitable positive (fungal culture positive corneal scraping with* Bipolaris* spp.,* Curvularia* spp.,* Exserohilum *spp., etc.) and negative controls. The PCR identified* Fusarium, A. flavus, *and* A. fumigatus* were subsequently determined to be culture positive. Though a prompt identification of fungal causative agent is the most important task behind every successful management of mycotic keratitis, the issue never has been completely redressed. The impediments of rapid identification could be possibly due to the less than a minimum quantity of the samples and cross contamination of conjunctival flora. Therefore, studies on rapid identification of fungi directly from corneal scrapings are very limited. Hence, the present study evaluated direct identification of major fungal agents such as* Fusarium *and* Aspergillus* directly from corneal scraping using multiplex PCR. The specificity of the PCR primers was determined by evaluation of positive control using known fungal isolates and negative control (bacterial DNA) and subsequent observation of culture positive cases. PCR confirmed positivity for all the positive control specimens while no bands in negative controls. The specimens positive for* Fusarium* spp. and* Aspergillus* spp. in PCR analyses were also identified to be culture positive in respective microbiological media.

Ferrer* et al.* 2001 [21] reported high specificity of PCR upon amplification of ITS of the fungal genome isolated from ocular infection when DNA isolated from human leukocytes and bacterial DNA were used as a negative control. Zunaina* et al.* 2008 [40] used 18S rRNA segment for direct identification of fungal pathogens from corneal scrapings, in which the specificity (94.7%) was confirmed by sequencing of amplified DNA fragment. The PCR sensitivity of the present study for* Fusarium* spp.,* A. flavus,* and* A. fumigatus* was 10 fg, 1 pg, and 300 pg/micro liter of DNA, respectively. Other investigators reported the sensitivity of* A. versicolor* genome with 100 fg [40]. In a significant difference from our findings,* A. fumigatus* sensitivity was also reported up to 1 fg upon semi-nested PCR [21]. A positive PCR detection of the fungal pathogens was reported by Emma et al. 2000 [41] from a single patient which was culture negative. However, in the present study, PCR detected and amplified fungal DNA from 10 patients. Ferrer* et al.* 2011 [42] asserted that fungal PCR must be added as the screening diagnosis since PCR not only proved to be an effective rapid method for the diagnosis of fungal keratitis but was also sensitive compared to staining and culture methods of investigation. To the best of the literature survey, the present study was the first attempt to identify the species of* Fusarium* and* Aspergillus* directly from corneal scrapings of culture proven fungal keratitis cases. The outcomes of the preliminary assessment were highly specific and sensitive in detecting* Fusarium* and* Aspergillus*. An extended scientific evaluation and optimization is being suggested as to apply PCR* in vitro* assays for a routine as well as rapid diagnostic applications.

## Figures and Tables

**Table 1:** Microbial etiologies of corneal ulcer isolates during the study period.

**Fungi**	**Incidence**	**Percentage (*n* = 411)**
*Fusarium* spp.	216	52.5
*A. flavus*	48	11.6
*A. fumigatus*	11	2.6
*A. terreus*	5	1.2
*A. niger*	3	0.7
*A. tamarii*	1	0.2
*Bipolaris* spp.	22	5.3
*Curvularia* spp.	12	2.9
*Exserohilum* spp.	11	2.6
*Cladosporium* spp.	4	0.9
*Aureobasidium* spp.	3	0.7
*Exophiala* spp.	2	0.4
*Lasiodiplodia *spp.	1	0.2
*Pseudallescheria* sp.	1	0.2
*C. albicans*	1	0.2
*Alternaria* sp.	1	0.2
*Scedosporium* spp.	1	0.2
*S. apiospermum*	1	0.2
Unidentified dematiaceous fungi (UID)	21	5.1
Unidentified hyaline fungi(UIH)	37	9

**Bacteria**		

*S. aureus*	1	0.2
*Pseudomonas* spp.	1	0.2
*Nocardia* spp.	1	0.2
CoNS	1	0.2
*S. viridans*	1	0.2
*Citrobacter* spp.	1	0.2

**Mixed infection**		

CoNS* + Fusarium* spp.	1	0.2
*S. pneumoniae + Bipolaris* spp.	1	0.2
*Citrobacter* spp.* + Bipolaris* spp.	1	0.2
**Total**	**411**	**100**

CoNS: coagulase negative *Staphylococcus* spp.

**Table 2:** Minimum inhibitory concentration (*μ*g/ml) of antifungal agents against *Fusarium* spp. (n=200).

**Amphotericin B**				
MIC range	≤0.5 *μ*g/ml	≥1 *μ*g/ml	MIC_50_	MIC_90_
8 – 0.125	77 (38.5%)	123 (61.5%)	1	1

**Natamycin**				
MIC range	≤8 *μ*g/ml	≥16 *μ*g/ml	MIC_50_	MIC_90_
64 – 2	70 (35%)	130 (65%)	16	32

**Itraconazole**				
MIC range	≤16 *μ*g/ml	≥32 *μ*g/ml	MIC_50_	MIC_90_
32 – 4	15 (7.5%)	185 (92.5%)	32	32

**Voriconazole**				
MIC range	≤4 *μ*g/ml	≥8 *μ*g/ml	MIC_50_	MIC_90_
8 – 1	101 (50.5%)	99 (49.5%)	4	8

**Econazole**				
MIC range	≤4 *μ*g/ml	≥8 *μ*g/ml	MIC_50_	MIC_90_
8 – 2	38 (19%)	162 (81%)	8	8

**Clotrimazole**				
MIC range	≤4 *μ*g/ml	≥8 *μ*g/ml	MIC_50_	MIC_90_
8 – 0.5	145 (72.5%)	55 (27.5%)	4	8

**Ketoconazole**				
MIC range	≤8 *μ*g/ml	≥16 *μ*g/ml	MIC_50_	MIC_90_
16 – 2	35 (17.5%)	165 (82.5%)	16	16

**Table 3:** Minimum inhibitory concentration (*μ*g/ml) of antifungal agents against *Aspergillus* spp.

**Amphotericin B**					

Isolates	MIC range	≤0.5 *μ*g/ml	≥1 *μ*g/ml	MIC_50_	MIC_90_

*A. flavus* (*n* = 47)	8 – 0.25	21 (44.7%)	26 (53.4%)	1	2
*A. fumigatus* (*n* = 11)	4 – 0.25	5 (45.4%)	6(54.5%)	1	4
*A. terreus* (*n* = 5)	2 – 0.5	2 (40%)	3 (60%)	1	2
*A. niger* (*n *= 3)	0.5 – 0.25	3 (100%)	-	0.5	0.5
*A. tamarii* (*n* = 1)	NA	1 (100%)	-	NA	NA

**Natamycin**					

Isolates	MIC range	≤16 *μ*g/ml	≥32 *μ*g/ml	MIC_50_	MIC_90_

*A. flavus* (*n* = 47)	64 – 16	18 (38.2%)	29 (61.7%)	32	64
*A. fumigatus* (*n* = 11)	64 – 16	7 (63.6%)	4 (36.3%)	16	32
*A. terreus* (*n* = 5)	32 – 16	1 (20%)	4 (80%)	32	32
*A. niger* (*n *= 3)	32 – 8	2 (66.7%)	1 (33.4%)	16	32
*A. tamarii* (*n* = 1)	NA	-	1 (100%)	NA	NA

**Itraconazole**					

Isolates	MIC range	≤0.25 *μ*g/ml	≥0.5 *μ*g/ml	MIC_50_	MIC_90_

*A. flavus* (*n* = 47)	1 – 0.25	25 (53.1%)	22 (46.8%)	0.25	0.5
*A. fumigatus* (*n* = 11)	0.5 – 0.25	5 (45.4%)	6 (54.5%)	0.5	0.5
*A. terreus* (*n* = 5)	NA	-	5 (100%)	0.5	0.5
*A. niger* (*n *= 3)	0.5 – 0.25	2 (66.7%)	1 (33.4%)	0.25	0.5
*A. tamarii* (*n* = 1)	NA	1 (100%)	-	NA	NA

**Voriconazole**					

Isolates	MIC range	≤0.5 *μ*g/ml	≥1 *μ*g/ml	MIC_50_	MIC_90_

*A. flavus* (*n* = 47)	4 – 0.25	37 (78.7%)	10 (21.2%)	0.5	1
*A. fumigatus* (*n* = 11)	4 – 0.25	6 (54.5%)	5 (45.4%)	0.5	1
*A. terreus* (*n* = 5)	1 – 0.5	3 (60%)	2 (40%)	0.5	1
*A. niger* (*n *= 3)	1 – 0.25	2 (66.7%)	1 (33.4%)	0.5	1
*A. tamarii* (*n* = 1)	NA	-	1 (100%)	NA	NA

**Econazole**					

Isolates	MIC range	≤0.5 *μ*g/ml	≥1 *μ*g/ml	MIC_50_	MIC_90_

*A. flavus* (*n* = 47)	2 – 0.25	37 (78.7%)	10 (21.2%)	0.5	1
*A. fumigatus* (*n* = 11)	1 – 0.25	5 (45.4%)	6 (54.5%)	1	1
*A. terreus* (*n* = 5)	1 – 0.5	2 (40%)	3 (60%)	0.5	1
*A. niger* (*n *= 3)	2 – 0.25	1 (33.4%)	2 (66.7%)	2	2
*A. tamarii* (*n* = 1)	NA	-	1 (100%)	NA	NA

**Clotrimazole**					

Isolates	MIC range	≤0.5 *μ*g/ml	≥1 *μ*g/ml	MIC_50_	MIC_90_

*A. flavus* (*n* = 47)	1 – 0.125	31 (65.9%)	16 (34%)	0.5	1
*A. fumigatus* (*n* = 11)	1 – 0.125	8 (72.7%)	3 (27.2%)	0.5	1
*A. terreus* (*n* = 5)	1 – 0.5	1 (20%)	4 (80%)	1	1
*A. niger* (*n *= 3)	1 – 0.5	1 (33.4%)	2 (66.7%)	1	1
*A. tamarii* (*n* = 1)	NA	1 (100%)	-	NA	NA

**Ketoconazole**					

Isolates	MIC range	≤0.5 *μ*g/ml	≥1 *μ*g/ml	MIC_50_	MIC_90_

*A. flavus* (*n* = 47)	8 – 0.5	14 (29.7%)	33 (70.2%)	1	4
*A. fumigatus* (*n* = 11)	4 – 0.125	4 (36.3%)	7 (63.6%)	1	2
*A. terreus* (*n* = 5)	4 – 1	-	5 (100%)	2	4
*A. niger* (*n *= 3)	4 – 0.5	1 (33.4%)	2 (66.7%)	1	4
*A. tamarii* (*n* = 1)	NA	-	1 (100%)	NA	NA

NA: not applicable.

## Data Availability

The data used to support the findings of this study are included within the article.
